# Understanding, fast and shallow: Individual differences in memory performance associated with cognitive load predict the illusion of explanatory depth

**DOI:** 10.3758/s13421-024-01616-6

**Published:** 2024-09-04

**Authors:** Christian Gaviria, Javier Corredor

**Affiliations:** Department of Psychology, Universidad Nacional de Colombia, Cr. 30 #45-03, Ed. 212, Of. 219, Bogotá, Colombia

**Keywords:** Metacognition, Explanation, Cognitive load, Social desirability, Illusion of Explanatory Depth

## Abstract

People are often overconfident about their ability to explain how everyday phenomena and artifacts work (devices, natural processes, historical events, etc.). However, the metacognitive mechanisms involved in this bias have not been fully elucidated. The aim of this study was to establish whether the ability to perform deliberate and analytic processes moderates the effect of informational cues such as the social desirability of knowledge on the Illusion of Explanatory Depth (IOED). To this purpose, the participants’ cognitive load was manipulated as they provided initial estimates of causal understanding of national historical events in the standard IOED paradigm. The results showed that neither the social desirability of specific causal knowledge nor the cognitive load manipulations had direct effects on the IOED. However, subsequent exploratory analyses indicated that high cognitive load was related to lower performance on concurrent memory tasks, which in turn was associated with a higher IOED magnitude. Higher analytical processing was also related to lower IOED. Implications for both dual-process models of metacognition and the design of task environments that help to reduce this bias are discussed.

## Introduction

Over the past decades, robust evidence has been collected showing that people overestimate the quality and accuracy of their knowledge and skills (Kruger & Dunning, 1999; Moore, 2020). This finding has been replicated across a wide range of tasks, such as recalling previously memorized information (Koriat & Bjork, 2005), answering general scientific knowledge questions (Lackner et al., 2023; Light et al., 2022), assessing text comprehension (Jaeger & Wiley, 2015), and making financial decisions (LaBorde et al., 2015). Moreover, subsequent studies have shown that the magnitude of this overconfidence may be greater in some types of knowledge, such as mechanistic explanations (Rozenblit & Keil, 2002). This phenomenon is known as the Illusion of Explanatory Depth (IOED, hereafter). Although this metacognitive bias has been studied in a variety of contexts and domains, there is no consensus on their causal mechanisms (Meyers et al., 2022). In this study, we hypothesized that the IOED may be related to the use of socially derived cues in the processes of estimating one’s knowledge, and that the ability to perform Type 2 processing moderates this relationship. If this hypothesis is correct, increasing the load on executive functions such as working memory may cause less accurate estimates of one’s causal knowledge.

### Illusion of explanatory depth

Rozenblit and Keil (2002) asked their participants to estimate their understanding of several phenomena and artifacts before and after writing detailed explanations of their mechanisms. Specifically, participants were taught on a 7-point scale to rate their understanding of a phenomenon, using examples of shallow, partial, and deep understanding. Then, participants rated their mechanistic understanding of how 48 items (e.g., can opener, zipper) work. In the third phase, participants wrote a step-by-step explanation of four of the items’ functioning, in which they were to present a causal chain connecting the steps. In the fourth step, participants solved a question that required understanding the items’ mechanism to be answered correctly, and, then, read an expert explanation of the mechanism. Finally, participants were asked to rate their mechanistic understanding of the items’ functioning on the same scale used in steps one and two.

Using different variations of this paradigm, Rozenblit and Keil found that participants reported a higher causal understanding before producing these explanations than after doing so. Importantly, the overestimation of understanding was higher for causal mechanisms than for knowledge about facts (e.g., national capitals), narratives (e.g., movie plots), or procedures (e.g., how to cook pasta). The IOED has been reported in both novice and expert adults (Fisher & Keil, 2016; Lawson, 2006) and children (Mills & Keil, 2004). Moreover, this phenomenon has been replicated in understanding ratings of diverse physical and social mechanisms, from everyday devices (Roeder & Nelson, 2015) to the consequences of policy proposals (Alter et al., 2010; Sloman & Vives, 2022).

The traditional explanation of the IOED suggests that it is a by-product of the intuitive theories used to understand the general causal structures of objects and phenomena, in which information about specific mechanisms is overlooked (Keil, 2012; Keil & Lockhart, 2021). According to this account, people confuse their ability to infer the domain of a mechanism from its general causal properties with the ability to explain in detail its functioning. For example, when people manage to recognize the global attributes of a phenomenon (e.g., which natural disasters are associated with climate change), they may mistakenly assume that they understand how it works (e.g., the relationship between climate change and the interactions between greenhouse gases and the infrared light, etc.; Ranney & Clark, 2016).

Although this approach is compatible with contemporary theories of knowledge acquisition and concept formation (e.g., Keil, 2022; Mahr & Csibra, 2022), it does not explain why people overestimate their understanding of some mechanisms more than others. For instance, those who have completed undergraduate studies overestimate their ability to explain topics in their area of training (e.g., the laws of thermodynamics or object-oriented programming) but not in everyday topics (e.g., how WiFi or birth control devices work; Fisher & Keil, 2016). This finding shows that the unspecific content of general intuitive theories is not the sole cause of the IOED. Instead, the knowledge that people have acquired throughout their life course contributes to exacerbating or diminishing the IOED about certain phenomena or artifacts.

### The role of social inferential cues in metacognitive processes

A plausible explanation for these differences in the IOED magnitudes is that they may be related to the influence of socially derived cues. In particular, when people estimate their understanding of a specific topic, they may not only take into account what they actually know, but also their perception of what they *should* know. This interpretation is consistent with some studies suggesting that people are more likely to overestimate the quality of their arguments on policy issues they consider more important (Fisher & Keil, 2014), even after controlling for the effect of the issue’s complexity and perceived familiarity (Johnson et al., 2017). On the other hand, people are more likely to overestimate their knowledge of a topic when they perceive that other members of their community have a good understanding of it (Rabb et al., 2020), or when they are told that it is part of their area of expertise (Ehrlinger & Dunning, 2003). In a more recent work, Gaviria & Corredor (2021) found that IOED is higher for topics for which explanatory knowledge is perceived as more socially desirable. In the first phase of these series of studies, either an independent sample or the actual participants rated the social desirability of causal knowledge about social and historical topics. Next, the topics for which knowledge was rated as either low or high in a social desirability scale were separated and assigned to different groups of participants in an IOED paradigm. The results revealed a positive association between the social desirability scores and the overestimation of explanatory knowledge (the IOED magnitude). Importantly, this effect held even after accounting for potential cofounding factors, such as familiarity and self-enhancement bias. Taken together, these previous works show that contextual and social factors other than the causal properties of mechanisms influence the magnitude of the IOED.

Consistent with the above, research on metamemory has shown that people make judgments about their own knowledge based on inferential cues, rather than on exhaustive recollection of long-term memories. For example, processing fluency, which is defined as “a subjective feeling of ease or difficulty associated with any type of mental processing” (Graf et al., 2018, p. 394), influences the judgments of learning or JOL (e.g., the estimated likelihood of recalling previously studied information in the future; see Bjork et al., 2013). Accordingly, it has been found that low processing fluency is typically associated with longer study times, lower JOL estimates, and lower recall accuracy (Koriat et al., 2006). In addition, two or more inferential cues may have interactive effects on metacognitive judgments. For example, the accuracy of text comprehension judgments depends on the accessibility of related information (Dunlosky, et al., 2005), but this effect may be conditional on the familiarity with the content (Koriat & Levy-Sadot, 2001). On the other hand, explicit beliefs about the influence of some contextual factors can modulate metacognitive judgments, just as inferential cues do. For example, when participants were led to believe that the color of words affected their visual processing, their JOL estimates were influenced by this feature, even though it did not have any real effect on retrieval accuracy (Mueller & Dunlosky, 2017). In summary, it has been proposed that metacognitive judgments are based on two kinds of inputs or sources: (1) feelings and experiences related to carrying out cognitive processes (e.g., processing fluency, accessibility, familiarity, etc.; Koriat & Levy-Sadot, 1999; Koriat et al., 2008), or (2) explicit information (e.g., beliefs related to one’s or others’ performance; e.g., Serra & Ariel, 2014). Supporting this hypothesis, empirical research has shown that both explicit information and feelings influence the magnitude of several metacognitive judgments (Ackerman & Thompson, 2015; Bjork et al., 2013). Importantly, if judgments made in metamemory tasks and judgments of causal understanding rely on similar mechanisms, the magnitude of the IOED might also vary as a function of social features attributed to specific explanatory knowledge.

On the other hand, some cognitive abilities could moderate the influence of social and motivational cues on metacognitive judgments. For example, Fernbach et al. (2013) found that the IOED tends to be lower when people can perform analytical processing and higher when they rely on automatic processing. Social cues, such as social desirability, are processed intuitively to a large extent (Miles et al., 2019; Schütz et al., 2020). Therefore, it is possible that changes in the level of analytical processing influence the effect of social cues, such as social desirability. In the following section, we explore the relationship between metacognition and dual processing.

### Dual processing theories and metacognition

Dual process models propose two information processing pathways. Type 1 processing is autonomous, fast, associative, based on contextual cues, uncontrolled, and has a low demand of cognitive resources. Type 2 processing, on the other hand, is analytical, sequential, slow, controlled, computationally demanding, and based on explicit rules (Kahneman, 2011; Pennycook, 2017). More specifically, Evans and Stanovich (2013) propose that Type 1 processing operates autonomously with respect to general purpose executive functions. That is, it does not require controlled attention to initiate and has a minimal demand on central executive resources (Baddeley et al., 2021). Therefore, increasing demand on executive resources is expected to affect people’s performance in tasks requiring analytical inferences. Consistent with this interpretation, previous studies have shown that manipulating working memory load through concurrent tasks substantially affects performance in several reasoning tasks (De Neys, 2006), including the Cognitive Reflection Test (CRT; Johnson et al., 2014). This task has been traditionally used in research on dual processes in reasoning, because it measures how much participants can inhibit erroneous intuitive responses and get the correct answer through controlled processing (Frederick, 2005).

The ability to process information analytically not only has effects on performance in specific tasks, but it is also associated with more general behaviors and dispositions. For example, people who have more difficulty making Type 2 inferences tend to exhibit a moral orientation more associated with social group identification (Pennycook et al., 2014), and accept fake news spread on social networks (Pennycook & Rand, 2020). Additionally, performance on the CRT is a more robust predictor of understanding scientific concepts and theories than the ability to analyze statistical information or knowledge about scientific methods (Shtulman & McCallum, 2014). In this sense, it is possible that Type 2 inferences are not only related to a better understanding of more complex concepts, but also to the ability to check the quality of that understanding. Supporting this idea, Fernbach et al. (2013) reported that people with high scores on the CRT showed a lower IOED regarding commercial products. In other words, higher scores on the CRT correlated with less overestimation of causal understanding. This finding is especially relevant for this study, because it shows that the ability to perform Type 2 processing is associated with greater accuracy in causal understanding judgments. As shown below, understanding the relationship between metacognitive accuracy and analytical processing requires considering the levels of organization of metacognitive processes.

### Inferential cues and levels of metacognitive processing

In the last decade, dual theories of processing have been extended from cognitive processing to metacognitive ones (Proust, 2012; Shea et al., 2014). In particular, Arango-Muñoz (2011) has distinguished between low- and high-level metacognition. Low-level processes depend only on the ability to have metacognitive sensations and form non-conceptual representations of cognitive processes (e.g., ease or speed). On the other hand, high-level processes additionally involve the ability to represent the mental states of other agents and to have an explicit knowledge base of how cognition works. Because of their declarative nature, high-level metacognitive processes are associated with Type 2 processing, whereas low-level processes are related to Type 1 inferences. Furthermore, both kinds of processes may interact (e.g., in some cases high-level processes may facilitate or inhibit the judgments generated by low-level processes).

The distinction between low- and high-level metacognition is compatible with the distinction between metacognitive judgments based on heuristic cues and on explicit information (Koriat et al., 2008). It is also consistent with the hypothesis that an inferential cue could affect several metacognitive judgments through different processing routes (Dunlosky & Tauber, 2014). Following this dual interpretation of metacognitive processes, social cues (such as social desirability) could affect the estimation of causal knowledge in at least two alternative ways: (1) through low-level processes that generate the metacognitive feeling of having more detailed knowledge about the most desirable topics, or (2) from high-level processes, in which the explicit belief that one is able to explain socially relevant phenomena influences the metacognitive experience associated with the topic.

How can we empirically establish which of these two processes connects the social desirability of knowledge with judgments of causal understanding? One methodological alternative is to use the two-response paradigm, created in the context of research on metacognition and reasoning (Thompson et al., 2011). This experimental paradigm is divided into two phases: first, participants are presented with a reasoning problem and asked to solve it as quickly as they can, so that they do not have enough time to make analytical inferences. In the second phase, participants have unlimited time to reflect on the problem and decide whether to modify their initial response. To prevent people from making "fast" analytical inferences before generating the first response, Bago & De Neys (2017) added to the initial phase a concurrent visuospatial memory task that increased the demand on central executive resources. An important advantage of this paradigm is that it allows intuitive responses to be clearly distinguished from analytical responses. Using an analogous principle, the IOED paradigm could be adapted to control for the level of metacognitive processes involved: if social desirability affects causal understanding judgments only through high-level metacognitive processes, it can be predicted that interfering with these processes through cognitive load and time-pressure manipulations should reduce the association between social desirability and the IOED. On the other hand, if social desirability influences judgments of causal understanding primarily through low-level processes, cognitive load or time-pressure manipulations should have a negligible effect on the association between social desirability of knowledge and the IOED magnitude. In this context, the present study aimed to establish whether social desirability of knowledge is associated with the IOED magnitude and whether restrictions on analytical inferences imposed by cognitive load have any effect on this association. Specifically, we attempted to test two related hypotheses:

- H_1_: The social desirability of a topic will be positively correlated with the magnitude of the IOED (replicating the findings of Gaviria & Corredor, 2021; see above).
- H_2_: The positive correlation between social desirability scores and the IOED will be higher for participants experiencing high cognitive load and time pressure compared to those with low cognitive load and no time pressure.

To empirically test these hypotheses, it is crucial to include a cognitive load task that may inhibit Type 2 processes. To demonstrate that accurate analytical inferences demand a larger amount of executive resources, Johnson et al. (2014) used a secondary task that required remembering three- or four-dot patterns in a 3 × 3 matrix (see *Materials and procedure* for details). Importantly, they found the response accuracy in tasks requiring Type 2 inferences is lower under high cognitive load (3%) compared to a low cognitive load condition (16%). Considering these results, the same visual task, along with a syllable memory task and a time-pressure factor, were used in the present study for the experimental manipulation of cognitive load.

We also included the CRT scores and prior participation in history courses as covariates. Given that these variables are introduced to control for possible confounds, they are not explicitly mentioned in H_1_ and H_2_. However, based on previous research, two hypotheses can be formulated about them. First, replicating the findings of Fernbach et al. (2013), CRT scores should correlate negatively with IOED, because they are related to a more vigilant processing during the understanding ratings. Second, students who had participated in history courses should have read and produced more explanations in this field and, therefore, their understanding ratings might be more accurate.

## Method

### Sample size

Johnson et al. (2016) used data from Rozenblit and Keil (2002; Studies 2 and 3) to estimate the number of participants required to observe the IOED in independent samples. Specifically, the means and standard deviations from these studies were employed to calculate the effect size of the IOED (Cohen’s *d* = 0.49). Using this parameter, a power analysis was performed to estimate the sample size required to detect a similar effect. The result was *n* = 64 per experimental condition, with a statistical power of 1-β = 0.80 and α = 0.05.

To confirm that this sample size was large enough to test the interaction between social desirability and cognitive load, an approximation of the effect size was obtained from the effect size of the joint effect of social desirability and processing type in Gaviria & Corredor (2021, p. 823). This decision was made because no previous experiment had examined the same combination of variables used in this experiment (social desirability and cognitive load). This interaction had an *r* of 0.29, which corresponds to Cohen's *d* = 0.61. The sample size required to test this interaction in a linear model was 84 participants, so the sample size used in this experiment was adequate to test the interaction.

The sample size calculations above were calculated to determine the sample size requirements for a linear model. However, because current replication standards require the introduction of stimuli as a random factor in models (Wolsiefer et al., 2017), the sample size calculation was double-checked for a mixed model. In this case, the standard procedure is to run a simulation (Kumle et al., 2021). As a result, a simulation was performed to determine the sample size required to detect the main effects of social desirability, cognitive load, and the interaction between these factors. The effect size of social desirability was estimated from the data of Gaviria & Corredor (2021, p. 820). The effect size of the cognitive load manipulation was assumed to be similar to that of social desirability, as there are no reported data on how this factor affects the IOED magnitude. A design with 256 participants (64 per cell) was found to have a statistical power above 0.85, 95% confidence interval (CI) [0.86, 0.90]. The simr package in R (Green & MacLeod, 2016) was used to run this simulation (the script is available in the Open Science Framework repository, see *Code availability*). According to this criterion, 60 or more participants per cell in the 2 × 2 design (4 × 60 = 240) were recruited in this experiment.

The total number of participants recruited was slightly larger than required by the power analysis (64 × 4 = 256), because it was expected that some participants might have to be excluded due to extremely low performance on the memory tasks. In fact, seven participants were excluded from the analysis because their scores on the visuospatial memory task and the attention check questions (see *Procedure*) were three standard deviations or more below the mean of their group. Thus, the sample size after exclusion was *n* = 255.

### Participants

Two hundred and sixty-two college students (164 women) participated in this experiment. Participants’ ages were between 18 and 50 years (*M* = 20.9 years, *SD* = 3.6 years). No history or economics students participated in this study. All participants gave written informed consent to participate in the study and most of them received academic or socio-economic support credit for their collaboration.

### Design

A 2 × 2 mixed experimental design was implemented. Social desirability of knowledge (high/low) and cognitive load (high/low) were manipulated as between-subjects factors, while the assigned topic was introduced as a random factor. This model specification follows recent recommendations to increase the replicability of psychological findings (Wolsiefer et al., 2017). In particular, factorial designs that ignore systematic variation in responses due to stimuli can produce significant results that cannot be replicated when different stimuli are used (Judd et al., 2012). Therefore, current literature recommends including the stimuli, tasks, or questions used in experiments as a random factor in addition to the fixed effects included in the factorial design (Asendorpf et al., 2013).

In the first phase of the experiment, each participant rated their causal understanding of each of the eight Colombian historical events or topics listed in Table 1. In the subsequent phases, however, they wrote an explanation and rated their understanding about only one of these events, which was randomly assigned. Because not all participants explained and re-rated their causal understanding of the same historical event, the assigned topic was introduced as a random factor, allowing the model to control for stimulus variability. It was decided that participants would explain only one historical event for two practical reasons. First, to keep the duration of the experiment within reasonable limits. Second, to avoid possible carryover effects due to repeated exposure to the IOED paradigm (see Roeder & Nelson, 2015).

**Table 1:** Illusion of Explanatory Depth (IOED) and social desirability ratings of historical events

Social desirability condition	Historical event	IOED		Social desirability ratings		Cronbach’s α (Social desirability scale)
		*M* (*SE*)	95% CI	*M* (*SE*)	95% CI	
Low	Alliances between politicians and paramilitary groups in the 1990s and 2000s	1.13 (.30)	[.52, 1.74]	4.68 (.22)	[4.22, 5.14]	.75
	Creation of the movement to change the Colombian Constitution in 1991	1.26 (.29)	[.67, 1.86]	4.47 (.25)	[3.95, 4.99]	.74
	Genocide of the Patriotic Union Party in the 1980s and 1990s	.89 (.22)	[.45, 1.34]	4.14 (.19)	[3.74, 4.53]	.77
	US government funding of the Plan Colombia in the 2000s	.81 (.21)	[.39, 1.24]	3.73 (.26)	[3.2, 4.25]	.72
High	Creation of FARC-EP guerrilla in the 1960s	1.57 (.24)	[1.07, 2.06]	5.22 (.27)	[4.67, 5.8]	.77
	Creation of paramilitary groups in the 1980s	.56 (.23)	[.09, 1.03]	4.43 (.21)	[3.99, 4.87]	.75
	Prolongation of the internal armed conflict	.52 (.32)	[-.14, 1.17]	4.64 (.16)	[4.3, 4.97]	.69
	Higher intensity of the armed conflict in rural areas	1.18 (.26)	[.65, 1.7]	4.8 (.21)	[4.37, 5.23]	.72

*M* mean, *SE* standard error, *CI* confidence interval of the mean

The specific historical events were selected because they showed significant differences in their social desirability ratings in a similar sample of participants (Gaviria & Corredor, 2021, Experiment 3). The dependent variable was the difference in causal understanding estimates before and after writing explanations on the assigned topic, which is the typical measure of the IOED (see Fisher & Keil, 2016). Covariates included the score of each participant in the CRT, the percentage of correct answers (hit rate) in the memory tasks used to induce cognitive load, and prior academic experience with courses related to national history. Covariates were included following recent recommendations in the analysis of randomized control trials and classic literature indicating that the inclusion of covariates increases the precision of model estimates and their replicability when they are related to the dependent variable (European Medicines Agency, 2015; Food and Drug Administration, 2023; Morris et al., 2022; Zhang et al., 2008).

### Materials and procedure

#### Visuospatial memory task (VMT)

**Fig. 1:**
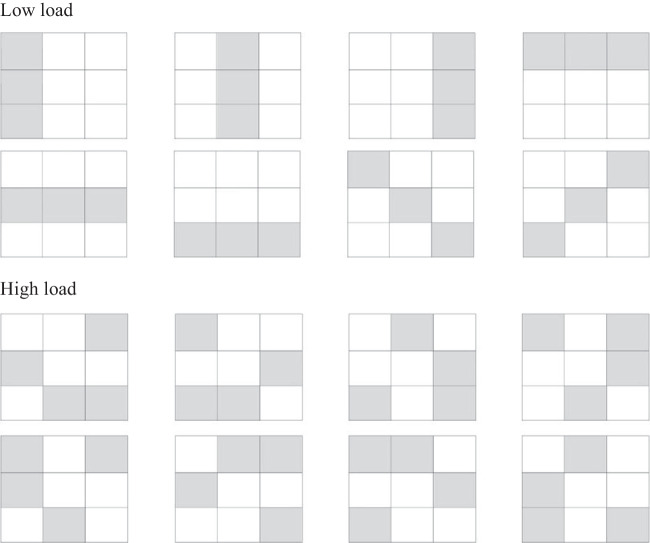
Matrices used in the visuospatial memory task in the low- and high-load conditions

Participants observed 3 × 3 matrices with colored boxes. In the low-load condition, three colored boxes formed horizontal or vertical lines. In the high-load condition, four boxes formed nonlinear patterns (see Fig. 1). These matrices were presented for 2 s. Later, participants reproduced the observed pattern by clicking on the corresponding boxes of a blank matrix. To avoid facilitation effects from repeated exposure, eight different arrays were used for each of the experimental conditions (see Fig. 1). This task was an adaptation of the dot memory task (De Neys, 2006). In previous studies, memorization of complex four-box patterns has been found to demand central executive resources (Miyake et al., 2001) and to interfere with performance on tasks requiring analytical inferences, such as the CRT (Johnson et al., 2014).

#### Syllable memorization task (SMT)

Participants were presented with two nonsense syllables for 2 s and asked to memorize them. To avoid practice effects, eight pairs of syllables were used. After performing the visuospatial memory task and the initial estimation of causal understanding for each historical event, they were asked to write the two syllables in the order in which they were displayed. The VMT and SMT tasks were included only in the high-load condition to increase the demand on both the visual and the verbal components of working memory (Baddeley et al., 2021).

#### Social desirability of knowledge scale

Four questions in a 7-point Likert scale were included for each of the historical events, which evaluated the social value and importance attributed to the understanding of such events. This scale included two positively (“how important is it for you to have a good understanding of…”; “how positive would other people’s opinion of you be, if you show a good understanding of…”) and two negatively framed questions (“how embarrassed do you think you would be if you do not know about…; “how negative would other people’s opinion of you be, if you do not show a good understanding of…”). The scale was identical to that previously used in Gaviria & Corredor (2021, Experiments 2–4). Total scores for each topic were calculated by averaging each participant’s responses to the four corresponding questions (see Table 1). The internal consistency index (Cronbach’s α) of the scale for each selected historical event is shown in Table 1.

#### Cognitive Reflection Test (CRT)

This three-item test measures the ability of individuals to perform analytical processing based on their responses to arithmetic problems that require suppressing a salient but incorrect intuitive response. The ability to answer the CRT correctly has been found to be associated with a general disposition for reflective thinking (Pennycook et al., 2015). The internal consistency of the CRT was moderate (Cronbach’s α = 0.63).

### Procedure

Participants performed the experimental tasks in computer cubicles in an isolated, noise-free room. After providing informed consent, participants answered the Instructional Manipulation Check (Oppenheimer et al., 2009) as a reading verification test. Next, participants were told how to perform the memory tasks and then solved two practice exercises for each task. If participants made any errors in the practice exercises, they were asked to reread the instructions and repeat the exercise. Answering this test correctly was a prerequisite for advancing to the main task. After this initial phase, participants received instructions on how to use the understanding scale.

When advancing to the main task, each participant was assigned to one of two experimental conditions: high or low cognitive load. Depending on the condition, they were asked to memorize certain stimuli (see above), which were presented for 2 s. Next, participants were given either 4 s (in the high-load condition) or unlimited time (in the low-load condition) to estimate their understanding of one of the historical events on a 7-point Likert scale. After making this judgment, they were asked to reproduce the stimulus presented in the memory task. As an attention check task, on the next screen, each participant was asked to identify the historical event the understanding of which they had just estimated from a list of several events. This sequence was repeated for each of the eight selected historical events, which were randomly presented (pre-explanation phase).

**Fig. 2:**
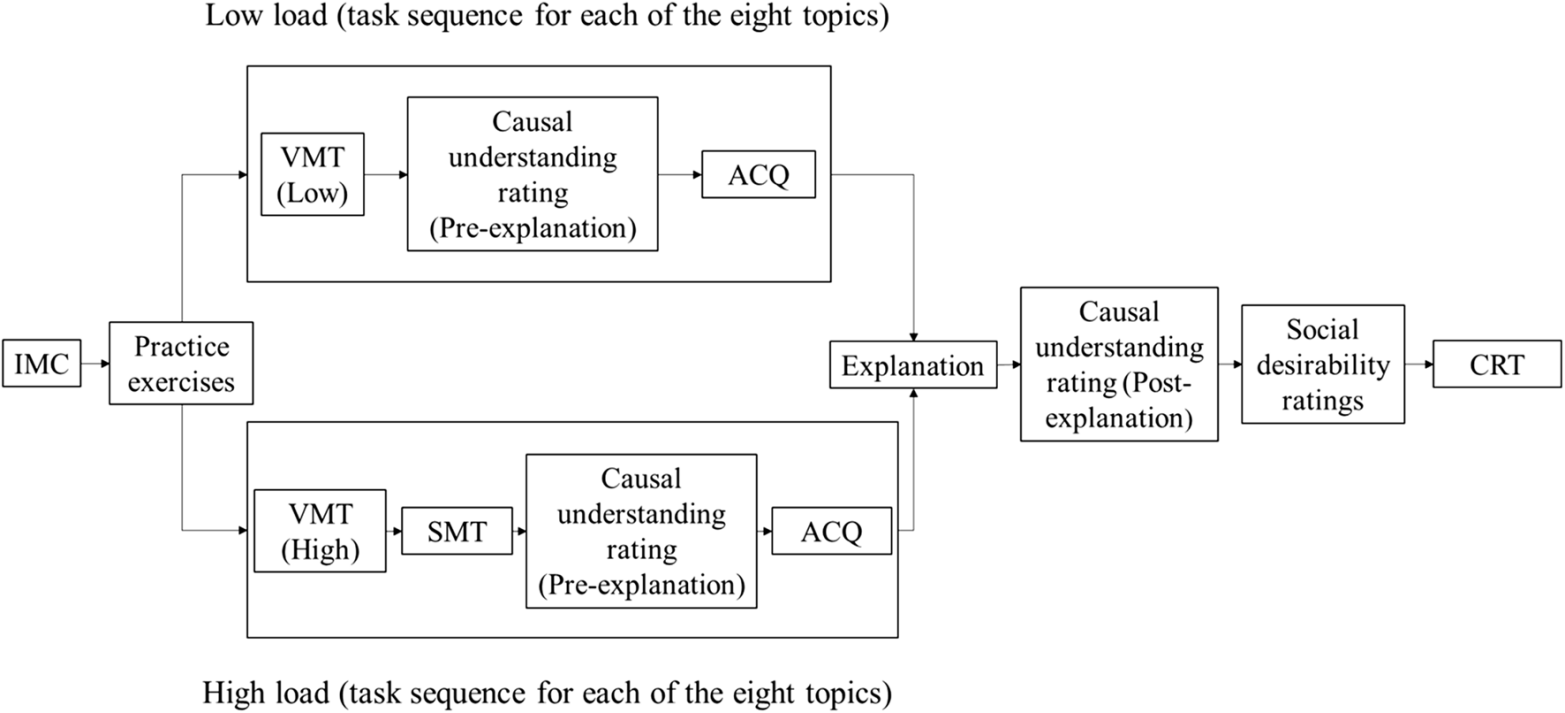
Flow diagram of the experimental tasks. *IMC* instructional manipulation check, *VMT* visuospatial memory task, *SMT* syllable memorization task, *ACQ* attention check questions, *CRT* Cognitive Reflection Test. See text for details

After completing their initial understanding judgments and memory tasks, participants were asked to write a step-by-step causal explanation of one historical event. This event could be a high or a low desirability topic, depending on the condition they were assigned to. After that, participants were asked to estimate their understanding of this topic again (post-explanation phase). Next, participants completed the social desirability scale for the eight historical events presented. Finally, participants answered the CRT and filled in the demographic information. Figure 2 shows the sequence of task presentation.

## Results

To establish whether the level of cognitive load influences the association between social desirability of knowledge and the IOED magnitude, hierarchical linear mixed models were built. In these models, the social desirability of the topic (high/low), the cognitive load (high/low), the CRT score, the hit rates in the memory tasks, and having taken courses on national history were included as fixed factors. The historical event explained by participants was added as a random effect (see *Design* section above).

### Hypothesis 1: IOED and social desirability of knowledge

Replicating the IOED, we found that the understanding ratings decreased after participants were asked to explain them, *M*_pre-post_ = 0.99, *SE* = 0.09, 95% CI [0.80, 1.17], *t*(254) = 10.51, *p* < 0.001, Cohen’s *d* = 0.66. Table 1 presents the differences between the pre and post estimates for each of the events in the main study. The IOED was found for all historical events in Colombia, regardless of the social desirability level, except for the prolongation of the internal armed conflict.

The social desirability condition was not a significant predictor of the IOED, χ^2^(2) = 0.11, *p* = 0.73 (see Table 2, Model 1), indicating that this manipulation did not have the expected effect. An explanation for this result is that individual differences in desirability were ignored in the group assignment (e.g., some participants attributed low desirability to an event belonging to the high-desirability condition or vice versa). Therefore, an alternative model was built in which the social desirability rating provided after completing the IOED paradigm was included as a predictor (posterior estimate). In this case, social desirability estimated by participants assigned to the high-desirability condition, *M* = 4.76, *SE* = 0.11, 95% CI [4.54, 4.98], was higher on average than that of the low-desirability group, *M* = 4.26, *SE* = 0.12, 95% CI [4.02, 4.49], *t*(250) = -3.08, *p* = 0.002, Cohen’s *d* = 0.39 (see Table 1). However, even in that model the association between the estimate of the social desirability of the assigned event and the IOED was not significant, χ^2^(4) = 2.99, *p* = 0.08.

**Table 2:** Hierarchical mixed linear models of the relationships between social desirability, cognitive load, and the Illusion of Explanatory Depth (IOED)

Model	Predictors	b (SE)	β	*t*	*df*	AIC	BIC
Model 0	Intercept	.99 (.12)	< .001	8.3	247	935.01	945.63
Model 1	Intercept	1.03 (.17)	.03	6.13	247	936.89	951.06
	Social desirability	-.08 (.24)	-.05	-.34	6		
Model 2	Intercept	.84 (.20)	-.01	4.23	246	935.3	953
	Social desirability	-.07 (.24)	-.04	-.27	6		
	Cognitive load	.35 (.19)	.24	1.9	246		
Model 3	Intercept	.87 (.22)	-.07	3.89	245	937.19	958.44
	Social desirability	-.13 (.31)	-.09	-.41	6		
	Cognitive load	.29 (.27)	.2	1.1	245		
	Social desirability * Cognitive load	.12 (.37)	.08	.33	245		
Model 4	Intercept	1.12 (.23)	-.1	4.81	244	925.91	950.70
	Social desirability	-.03. (31)	-.02	-.11	6		
	Cognitive load	.4 (.26)	.27	1.56	244		
	Social desirability * Cognitive load	-.16 (.37)	-.11	-.43	244		
	CRT scores	-.35 (.10)	-.23	-3.66***	244		
Model 5	Intercept	1.29 (.23)	.04	5.51	243	919.75	948.08
	Social desirability	-.03 (.3)	-.02	-.09	6		
	Cognitive load	.44 (.26)	.29	1.69	243		
	Social desirability * Cognitive Load	-.23 (.37)	-.2	-.64	243		
	CRT scores	-.32 (.10)	-.35	-3.28**	243		
	History courses	-.53 (.19)	-.16	-2.85**	243		

*CRT* Cognitive Reflection Test, *df* degrees of freedom. * *p* < .05, ** *p* < .01, *** *p* < .001

### Hypothesis 2: IOED, cognitive load, and social desirability of knowledge

Although a direct effect of social desirability on the IOED was not found, it is still possible that this factor interacts with the level of cognitive load (as specified in H_2_). To test this hypothesis, two additional models were built that included the cognitive load condition (see Table 2, Model 2) and the interaction term between social desirability and cognitive load (see Table 2, Model 3) as fixed factors. The associations between either of these factors and the IOED were not statistically significant in these models. Overall, the above results indicate that Gaviria and Corredor’s findings (2021, Experiment 3) were not replicated and neither H_1_ nor H_2_ (see *Introduction*) were supported by these data. That is, it is not possible to claim that participants overestimated their understanding of historical events more in the high cognitive load condition or when they judged the knowledge of such events as more socially desirable.

#### Exploratory analysis with measured variables

##### CRT scores and prior formal instruction

In the confirmatory analyses described above, there was no evidence of either a direct effect or an interactive effect of social desirability and cognitive load. However, it is possible that other measured variables had effects on the IOED. For instance, the ability to make Type 2 inferences (as measured by the CRT scores) may affect both performance on the memory tasks and the IOED magnitude. Similarly, prior knowledge acquired in history courses could both reduce the memory pressure and modify the understanding ratings during the IOED. In this vein, two additional models were built to test whether both CRT scores and having taken history courses had effects on the IOED magnitude (see Table 2, Models 4 and 5). We also checked that the included covariates were not mediators or colliders, according to Rohrer’s (2018) recommendations.

Replicating the finding of Fernbach et al. (2013), a negative association was found between CRT scores and the IOED, χ^2^(7) = 13.28, *p* < 0.001 (see Table 2, Model 4). That is, those who performed better on this test estimated more accurately their causal knowledge of the assigned historical event, regardless of the level of cognitive load. Finally, those who had taken or were taking university courses on Colombian history showed a lower IOED, χ^2^(8) = 8.16, *p* < 0.01, (see Table 2, Model 5). In short, both higher CRT scores and not having taken courses on national history were associated with higher IOED.

##### Individual performance in working memory tasks

The effect of the experimental condition manipulating cognitive load on the IOED was not significant, χ^2^(5) = 3.59, *p* = 0.06 (see Table 2, Model 2). However, as in the case of social desirability, this may have occurred because this dichotomous variable does not provide information about individual differences in the level of load induced by the assigned memory tasks. Thus, a participant in the low-load condition could have experienced a similar level of cognitive load as someone assigned to the high-load condition, because of differences in her or his working memory capacity. In other words, what matters is not only the cognitive load condition but the effects of this condition on executive functions.

To obtain an indicator of individual performance on the memory tasks, we averaged each participant’s hit rates on both the visuospatial memory task and the attention verification questions during the pre-estimation phase. If the experimental manipulation of cognitive load was effective, one would expect performance on these tasks to be higher in the low-load group. Corroborating this hypothesis, it was found that the hit rate on the memory tests was higher in the low-load condition, *M* = 0.97, *SE* = 0.01, 95% CI [0.95; 0.98], compared to the high-load group, *M* = 0.82, *SE* = 0.01, 95% CI [0.80; 0.84], *t*(192) = 12.8, Cohen’s *d* = 1.59, *p* < 0.001. Although this result does not provide an independent measure of cognitive load, it could be interpreted as an indirect check (see Ejelöv & Luke, 2020) of the cognitive load manipulation. This is because performance on this visuospatial task has previously been found to be correlated with executive functioning (De Neys, 2006; Miyake et al., 2001). Additionally, performance in memory tasks was negatively associated with the IOED magnitude both in the low cognitive load, *r* (124) = -0.23, *t* = -2.59, *p* = 0.01, 95% CI [-0.39; -0.05], and in the high-cognitive load group, *r* (127) = -0.26, -3.05, *p* = 0.002, 95% CI [-0.41; -0.09]. This result shows that variation in individual responses to the memory tasks is negatively correlated with IOED within and across conditions, which implies that responses to the conditions vary among individuals. In other words, the IOED was lower in participants who performed better on the working memory tasks, which was more prevalent in the low-load condition.

##### Exploratory mediation analysis

**Fig. 3:**
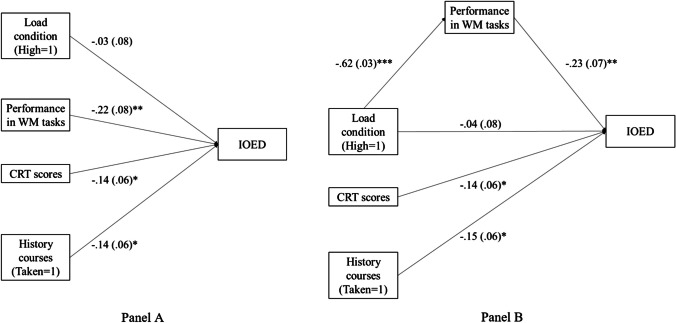
Structural equation models of the direct effect (**A**) and the mediated effect (**B**) of cognitive load and performance in memory. CRT = Cognitive Reflection Test, WM = Working memory, *IOED* = Illusion of Explanatory Depth. Values represent standardized coefficients with standard errors between parentheses; * *p* < .05, ** *p* < .01, *** *p* < .001

Given the above results, it is possible that the relationship between the cognitive load manipulation and the IOED magnitude was mediated by individual differences in working memory performance. To explore this alternative, two structural equation models (Gana & Broc, 2019) were built using the lavaan package in R (Rosseel, 2012). In the first model, the cognitive load condition and performance in memory tasks were included separately as predictors of the IOED (see Fig. 3, Panel A). In the second model, the effect of the load condition on the IOED is mediated by the performance in memory tasks (see Fig. 3, Panel B). In the initial model, a significant association was found between performance in the memory tasks and the IOED, β = -0.22, *p* = 0.004, 95% CI [-0.38; -0.07], but not between the cognitive load condition and the IOED, β = -0.03, *p* = 0.65. In the second model, a positive relationship was found between the load condition (high = 1, low = 0) and the magnitude of the IOED, which was fully mediated by performance on the memory tasks, β = 0.14, *p* = 0.003, 95% CI [0.05; 0.23]. That is, all significant effects of load condition go through changes in performance in memory tasks. The high-load condition decreases performance in memory tasks and decreased performance in memory tasks produces a higher IOED. The fit indices of the mediation model (AIC = 415.81; BIC = 440.6; CFI = 0.85; RMSEA = 0.23; SRMR = 0.07) were better than those of the direct effects model (AIC = 908.9; BIC = 926.7; CFI = 1; RMSEA = 0; SRMR < 0.001). Importantly, these differences suggest that including the effect of load condition on the performance in working memory tasks improves both the goodness of fit and the parsimony of the structural model.

## Discussion

In this experiment, we manipulated the level of cognitive load while participants judged their causal understanding about historical events whose knowledge had a high or low social desirability. This was done to establish whether limiting analytical processing during metacognitive judgments would change the effect of this social cue on such judgments. However, no direct or interactive effects of social desirability on the IOED were found. To explain this unexpected result, there are at least three possibilities, which are discussed in the next paragraph.

First, the differences between the social desirability scores of the items in each condition (low and high desirability) could be not large enough to allow for a proper between-groups comparison. Indeed, they were smaller (Cohen’s *d* = 0.39; see *Results*) than those found in a previous study using the same scale and events on a similar sample of participants (Cohen’s *d* = 0.97; Gaviria & Corredor, 2021, p. 818). Thus, the social desirability of knowledge about historical events may be a difficult variable to manipulate, because it is highly unstable over time and sensitive to small changes in the socio-political context (Wagner-Pacifici, 2017). In this vein, future experiments should include more contrastive sets of events in the low- and high-desirability conditions. Second, participants completed the social desirability scale immediately after they did the IOED paradigm. By doing so, the recent experience of success or failure explaining a particular event could have influenced the social desirability judgments. However, it might be also argued that the reverse order (asking for social desirability ratings at the beginning of the experiment) could have affected the initial understanding ratings in the IOED task. To solve this dilemma, the order of presentation of the social desirability scale and the IOED paradigm should be counterbalanced in future studies. Third, participants could have been induced to perform analytical processing even at the low-load level, due to the evaluative context generated by the memory tasks that were introduced in both conditions. Specifically, participants in both groups had to remember visual stimuli to ensure that all of them performed tasks of similar duration and to control for possible fatigue effects (see Johnson et al., 2014). Thus, the experimental manipulation could have created a marginal amount of cognitive load that restricted the use of social desirability as an informational cue in both experimental groups. Future research should include both a group of zero load, in which participants do not perform memory tasks during the initial estimation, and a high-load group, in which participants solve tasks equal or similar to those included in this experiment. Remarkably, even if the social desirability of knowledge does not have a robust effect on the IOED (e.g., if both H_1_ and H_2_ are false; see *Introduction*), the present results still show evidence of a negative correlation between working memory performance and the IOED magnitude, which is addressed below.

### Cognitive load and metacognitive biases

We found the cognitive load manipulation did not have uniform effects on causal understanding judgments. Instead, these were mediated by how well participants performed the concurrent memory tasks. In other words, cognitive load manipulations had differential effects depending on how participants with different performances in cognitive functions reacted to these manipulations (Engin, 2021). Importantly, this effect held even after controlling for the CRT scores. That is, the effect of the working memory performance on the IOED cannot only be attributed to differences in the ability to perform Type 2 inferences. Yet, there is evidence that analytical thinking and executive function are not fully independent mechanisms (Evans & Stanovich, 2013; Otero et al., 2022). In this vein, it remains unclear whether the negative correlation between working memory performance and the magnitude of the IOED found in this study is due to individual differences in working memory capacity, the effect of the cognitive load manipulation on executive resources, or a combination of both. This is because (1) potential confounding variables, such as the individual differences in working memory capacity, were not directly measured, and (2) the performance on working memory tasks (the mediator variable) was not randomized (Rohrer et al., 2022). Thus, although the cognitive load (the independent variable) was randomized to prevent any exogenous variable from confounding the relationship between the independent variable and the mediator variable, the results of the mediation analysis are only correlational and exploratory (MacKinnon, 2008). Future studies may help to overcome these limitations by (1) including standardized measures of working memory capacity and other executive functions as covariates, and (2) using causal estimation techniques, such as instrumental variable analyses, to address potential endogeneity problems (Gennetian et al., 2005).

There are several possible explanations for why performance on memory tasks in both load conditions was negatively associated with the magnitude of the IOED. For example, when people are asked to estimate their causal understanding of a given topic, they will try to recall information about it. However, explanatory knowledge is not likely retrieved as finished chunks of information from long-term memory. Instead, it is built "on the fly," using the most salient or accessible information at the time of elaboration, such as inherent or extrinsic features (Horne, et al., 2019). Thus, when a secondary task or time pressure interferes with this recall process, the estimation of causal understanding may be less accurate depending on both the amount and type of information being recalled. In this vein, future research could explore whether the IOED magnitude depends on content factors such as the easiness of retrieval of facts (inherent and extrinsic) about the object or phenomenon to be explained (Hussak & Cimpian, 2018).

An alternative explanation for the present findings is that the feeling of ease in the visual and syllable memorization tasks could have served as a heuristic cue for the evaluation of understanding in the IOED ratings. That is, it is possible that the feelings derived from the low-load manipulation spilled over to the metacognitive judgments. The memory tasks were contingent only to the first understanding rating. So, if an easier processing of the memory tasks is confused with a higher understanding of the topic, the prior understanding score would be higher for participants performing better in the memory tasks and the IOED would be lower for this group. This possibility needs to be explored in future research, for instance, by extending the memory tasks over both the prior and the posterior understanding ratings, using covariates to control how difficult memory tasks feel, or comparing understanding ratings with and without contingent memory tasks.

### Relevance and practical implications

This study provides evidence that the IOED magnitude depends not only on the representational features of causal knowledge (Alter et al., 2010) or beliefs about one’s knowledge (Fisher & Keil, 2016), but also on the executive resources available to assess one’s understanding. This finding has at least two general implications. First, it shows that some experimental paradigms from research on dual processes (such as the two-response paradigm) can be adapted to test hypotheses about interactions between low- and high-level processes in metacognitive judgments. Second, it suggests the possibility of reducing the magnitude of metacognitive biases, such as the IOED, by creating learning or deliberation environments in which people with lower working memory capacity have manageable demands on their central executive resources.

In this line, the theoretical relevance of this study comes from several points. First, it shows that dual-processing models proposed for cognitive activity (e.g., Pennycook, 2017) are also relevant for understanding metacognitive processes. The effect of working memory performance produced by the cognitive load manipulation shows that the availability of central executive resources plays a role in the production of metacognitive judgments. This connection between cognitive and metacognitive dual processes opens a promising line of research. Second, the study underscores the role of subjects’ mediational responses to experimental manipulations in cognitive studies. In general, it is assumed that manipulations guarantee subjects’ responses. However, as shown in this study, individuals respond in different ways to treatments, and those differences need to be accounted for. This type of logic has been explored extensively in economics and health sciences, and methodologies to control for them have been developed. For instance, instrumental variable estimation allows accounting for adherence (or lack thereof) to treatments, while producing causal estimates (Angrist, 2022). This study shows the relevance of incorporating these methodologies to psychology and cognitive research. Finally, this study highlights the importance of considering individual differences in the study of metacognitive processes, in this case, through the identification of working memory performance as a predictor of IOED. In a more general light, this study shows the relevance of studying metacognitive biases and the IOED in phenomena associated with history and social sciences.

The IOED and other metacognitive biases vary across individuals and need to be diagnosed in populations. Therefore, this study shows the need to develop appropriate measuring tools for these phenomena. The practical implications of this study also include the need to train individuals for controlled processing as a way to prevent the effects of metacognitive biases, especially in controversial cognition environments such as history, politics, or social issues. In the same vein, educational process to teach students to know and identify these biases are key to preventing them in many social spaces. The fact that memory performance derived from cognitive load influences the IOED also indicates the need to promote the creation of low cognitive social spaces for decision making. For instance, prior literature has shown that scarcity situations increase cognitive load and decrease cognitive bandwidth when making decisions (González-Arango et al., 2022). In this line, public systems need to support adequate metacognitive assessment by creating spaces where scarcity is, at least temporally, prevented, (e.g., well-stocked community centers or food banks). Also, the promotion of using low cognitive load spaces to make decisions should be a public issue (e.g., not double tasking on the cellphone while thinking about social issues).

This study also shows that people who were taking (or had taken) courses on national history estimated with greater accuracy their understanding of the assigned topics. This fact highlights the need to control more rigorously the level of prior knowledge of the participants in the IOED paradigm. Although people with more formal education are more liable to overestimate their causal knowledge about their areas of expertise (Fisher & Keil, 2016; Gutierrez & Montoya, 2021), it is possible that those who have recently had to provide elaborations on similar topics as part of their academic or professional activities may be able to make more accurate understanding judgments. In this sense, this article also points out the importance of promoting a Socratic approach, if the term is allowed, to political understanding, in which people are asked to explain their beliefs before making decisions about core issues.

## Data Availability

The dataset and materials generated during the current study are available in the Open Science Framework repository at: https://osf.io/c3vft/

## Code availability

All analyses were performed using the R environment. The scripts are available in the Open Science Framework repository at: https://osf.io/c3vft/
